# Renal tubular epithelial cells treated with calcium oxalate up-regulate S100A8 and S100A9 expression in M1-polarized macrophages via interleukin 6

**DOI:** 10.22038/IJBMS.2023.69202.15080

**Published:** 2023

**Authors:** Qing Wang, Jianqing Zhang, Xiaolong Chen, Fa Sun, Kehua Jiang

**Affiliations:** 1 Department of Urology, Guizhou Provincial People’s Hospital, Guiyang, Guizhou, 550000, China; 2 Zunyi Medical University, Zunyi, Guizhou, 550000, China

**Keywords:** Calcium oxalate, Interleukin 6, Nephrolithiasis, S100A8 protein, S100A9 protein

## Abstract

**Objective(s):**

Calgranulins S100A8 and S100A9 are common in renal stones and they are up-regulated in both urinary exosomes and kidneys of stone patients. Renal sources and important regulators for S100A8 and S100A9 in nephrolithiasis were explored in this study.

**Materials and Methods:**

We identified S100A8 and S100A9 abundance in various renal cells by searching the Single Cell Type Atlas. Macrophages were polarized from human myeloid leukemia mononuclear cells. Human proximal renal tubular epithelial cells (HK-2) were stimulated with calcium oxalate monohydrate (COM). Coculture experiments involving HK-2 cells and macrophages were conducted. qPCR, Western blotting, ELISA, and immunofluorescence were used for detecting interleukin 6 (IL6), S100A8, and S100A9.

**Results:**

The Single Cell Type Atlas showed that S100A8 and S100A9 in human kidneys primarily originated from macrophages. M1 was the predominant macrophage type expressing S100A8 and S100A9. Direct culture with COM did not affect the expression of these two calgranulins in M1 macrophages but coculture with COM-treated HK-2 cells did. COM could promote HK-2 cells to secrete IL6. IL6 could up-regulate S100A8 and S100A9 expression in macrophages of M1 type. In addition, 0.5 μM SC144 (a kind of IL6 inhibitor) significantly prevented COM-treated HK-2 cells from up-regulating S100A8 and S100A9 expression in macrophages of M1 type.

**Conclusion:**

M1-polarized macrophages were the predominant cell type expressing S100A8 and S100A9 in the kidneys of nephrolithiasis patients. CaOx crystals can promote renal tubular epithelial cells to secrete IL6 to up-regulate S100A8 and S100A9 expression in macrophages of M1 type.

## Introduction

Urolithiasis is a common public health problem and often causes renal colic, urinary tract infections, and renal function damage (1). 1%-15% of individuals may suffer from nephrolithiasis during their lifetime (2). Mechanisms underlying the stone formation remain unclear and this leads to the slow development of medical prevention over the past 30 years (3). The five-year recurrence rate for kidney stones after surgical intervention can be up to 50% (4), which brings huge morbidity and financial burden to society (5). Therefore, exploring the pathogenesis of nephrolithiasis is crucial for its treatment and prevention. 

Eighty percent of kidney stones are predominantly made up of calcium oxalate (CaOx) with different amounts of calcium phosphate (3). Our previous systematic analysis demonstrated that calgranulins S100A8 and S100A9 were the most frequently identified proteins in the renal stone matrix. Their expression was significantly elevated in the renal interstitium of CaOx stone patients (6). In addition, we also found that urinary exosomes from CaOx stone patients contained more S100A8 and S100A9 than those from healthy controls (7). These findings indicate that S100A8 and S100A9 might serve as both valuable therapeutic targets and biomarkers for kidney stone disease. To further study the role of S100A8 and S100A9 in nephrolithiasis, we think two important questions should be addressed first. One is where renal S100A8 and S100A9 originate, the other is how S100A8 and S100A9 expression in the kidneys of CaOx stone patients is regulated. 

In order to explore renal sources and important regulators for S100A8 and S100A9 in nephrolithiasis, the Single Cell Type Atlas (www.proteinatlas.org/celltype) was searched. In addition, macrophages differentiated from human myeloid leukemia mononuclear cells (THP-1) were cocultured with human renal tubular epithelial cells (HK-2) treated with calcium oxalate monohydrate (COM) to simulate the microenvironment of stone formation *in vitro*. 

## Materials and Methods

***Cell culture***

THP-1 and HK-2 cells were obtained from the Chinese National Collection of Authenticated Cell Cultures (Shanghai, China). Cells were cultured in a 37 ^°^C humidified environment with 5% CO_2_. For cell coculture experiments, transwell chambers (Corning, USA) were used. HK-2 cells with or without COM crystals were cultured in the upper chamber, and macrophages differentiated from THP-1 cells were cultured in the lower chamber.

***Macrophage polarization***

THP-1 cells were polarized into M0 macrophages by incubation with 200 ng/ml phorbol 12-myristate 13-acetate (MCE, USA) for 24 hr. To obtain M1-polarized macrophages, M0 macrophages were cultured with 100 ng/ml lipopolysaccharide (Sigma, USA) plus 20 ng/ml interferon-γ (MCE, USA) for 48 hr. To obtain M2 polarized macrophages, we cultured M0 cells with 20 ng/ml interleukin 4 (MCE, USA) plus 20 ng/ml interleukin 13 (MCE, USA) for 72 hr.

***Preparation of COM crystals***

COM crystals were prepared as described by Sakdithep *et al*. (8). Briefly, we mixed 5 mM CaCl_2_·2H_2_O and 0.5 mM Na_2_C_2_O_4_ with a buffer containing 10 mM NaCl and 90 mM Tris-HCl. After being incubated at 25 ^°^C overnight, the solution was centrifugated at 2000 g for 10 min. COM precipitate was then resuspended with the medium. The final dose of COM for culturing cells was 67 μg/cm^2^ according to previous studies (9-11). 

***Preparation of SC144 and human recombinant interleukin 6***

SC144 (MCE, USA) is an inhibitor of interleukin 6 (IL6), it was dissolved in dimethyl sulfoxide (DMSO) (Solarbio, China) for use. Recombinant human IL6 was purchased from Novoprotein (Soochow, China) and dissolved in phosphate-buffered saline for use. 

***Real-time quantitative polymerase chain reaction (qPCR)***

Cellular RNA was extracted with Trizol (Invitrogen, USA). Reverse transcription reactions were conducted with cDNA Synthesis SuperMix (Yeason, China). qPCR was conducted with SYBR Green Master Mix (Yeason, China) in a CFX Connect^TM ^system (Bio-Rad, USA). Primers in the current study were designed via PrimerBank (https://pga.mgh.harvard.edu/primerbank/) (12) and sequences were listed in Table 1. Sangon Biotech (Shanghai, China) was responsible for synthesizing all primers. We used Glyceraldehyde 3-phosphate dehydrogenase (GAPDH) as the reference gene. The 2^-∆∆Ct^ method was used to analyze the results. 

***Western blotting***

We extracted cellular protein with radioimmunoprecipitation assay buffer (Solarbio, China) and phenylmethanesulfonyl fluoride (Solarbio, China). Bicinchoninic acid protein assay kits (Solarbio, China) were used to analyze the protein concentration. Equivalent proteins from different samples were isolated by 12% gel electrophoresis. After being transferred to membranes, bands were blocked with 4% skimmed milk at room temperature for 1 hr and incubated with primary antibodies at 4 ^°^C overnight: rabbit monoclonal β-actin (Abclonal, China, 1:5000), rabbit monoclonal anti-S100A9 (CST, USA, 1:1000), rabbit polyclonal anti-S100A8 (Abclonal, China, 1:1000), and rabbit polyclonal anti-IL6 (Abclonal, China, 1:1000). Protein bands were then incubated in secondary antibodies (Abclonal, China, 1:5000) at room temperature for 1 hr. Chemiluminescence assay kits (NCM Biotech, China) were used to visualize the protein bands in Genegenome XRQ System (Syngene, Britain). ImageJ software (v1.8.0) was used to measure the density of protein bands and to convert the results into quantitative data.

***Immunofluorescence***

After being cocultured with HK-2 cells, M1-polarized macrophages were fixed in 4% paraformaldehyde for 20 min and permeabilized with 0.1% Triton X-100 for 30 min. Then cells were blocked in 5% bovine serum albumin for 2 hr. S100A9 (CST, USA, 1:100) or S100A8 antibody (Abclonal, China, 1:100) was used to incubate cells at 4 ^°^C overnight. Then cells were incubated with DyLight 549 or DyLight 488 IgG (Abbkine, USA, 1:200) for 1 hr. 4,6-diamino-2-phenyl indole (DAPI) (Beyotime Institute of Biotechnology, China) was used to stain nuclei for 10 min. Finally, S100A8 and S100A9 expressions were observed using a fluorescence microscope (Olympus, USA). 

***Enzyme-linked immunosorbent assay (ELISA)***

The cellular supernatant IL6 level was measured using an ELISA kit (Elabscience Biotechnology, China). Samples (100 μl) were added to wells and incubated at 37 ^°^C for 90 min. The liquid was then discarded and 100 μl biotinylated detection solution was immediately added and incubated at 37 ^°^C for 60 min. The liquid was then discarded and the plate was washed three times. 100 μl horseradish peroxidase conjugate solution was added and incubated at 37 ^°^C for 30 min. The liquid was then discarded and the plate was washed five times. 90 μl substrate reagent was added and incubated at 37 ^°^C for 60 min. Finally, 50 μl stop solution was added and the plate was read at 450 nm.

***Detection of cellular viability***

Cellular viability was detected with a cell counting kit-8 (CCK-8) (APExBIO, USA). M1-polarized macrophages were seeded in 96-well plates at a concentration of 3x10^3^/well and incubated overnight. They were treated with SC144 (0, 0.1 μM, 0.2 μM, 0.5 μM, 1 μM, and 2 μM) or dimethyl sulfoxide (2‰) for 48 hr. Then the medium was discarded and 90 μl Roswell Park Memorial Institute Medium containing 10 μl CCK-8 solution was added. After incubation for another 2 hr, the plate was read at 450 nm. Cellular viability was calculated as the percentage relative to the control. 

***Statistical analysis***

We repeated each experiment at least 3 times. SPSS-v25.0 (SPSS, USA) was used. Measurement data are presented as means±standard deviations. ANOVA or Student’s t-test was conducted for statistical analysis. *P*<0.05 was considered significantly different in the current study.

## Results

***M1-polarized macrophages were the predominant origin of S100A8 and S100A9 in renal tissue***

**Figure 1:**
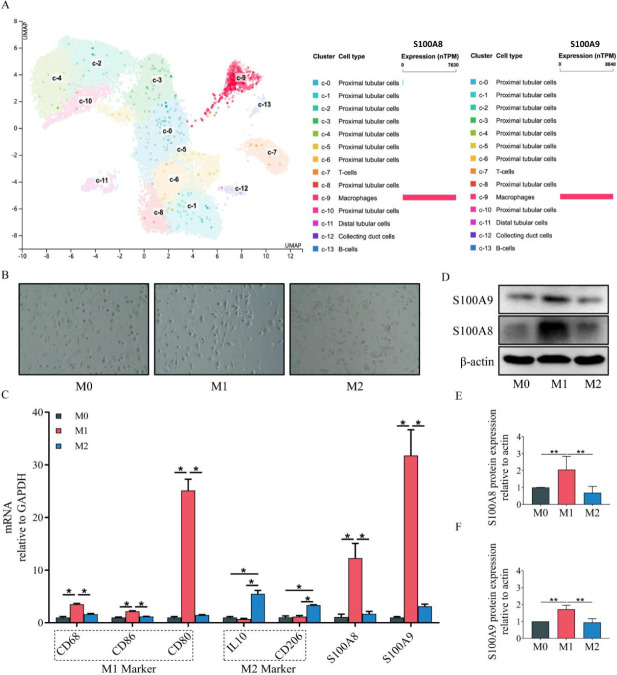
Origin of renal S100A8 and S100A9. (A) There were 14 cell-type clusters in the normal human kidney. Among all cell types, macrophages had the highest expression of both S100A8 and S100A9 (this figure was created with Single Cell Type Atlas). (B) Microscopic morphology of M0, M1, and M2 macrophages. (C) mRNA expression of M1 biomarkers (CD68, CD86, and CD80), M2 biomarkers (IL10 and CD206), S100A8, and S100A9 in different types of macrophages. (D-F) Western blotting showed that S100A8 and S100A9 expression in M1-polarized macrophages was much higher than that in M0 and M2 macrophages. (TPM: Transcripts per million, UMAP: uniform manifold approximation and projection, *P<0.05, **P<0.01)

Renal tissue consists of many cell types. Detecting S100A8 and S100A9 abundance in various cells is important for exploring the renal origination of these two proteins. The Single Cell Type Atlas is an open-access database that provides a single-cell map of 25 human tissue types and allows researchers to explore gene expression in specific cell clusters (13). Therefore, we searched the above database to explore the predominant cell type which expresses S100A8 and S100A9 in the kidney. As shown in Figure 1A from the Single Cell Type Atlas, there are various proximal tubular cells, distal tubular cells, collecting duct cells, macrophages, T cells, and B cells in normal renal tissue. Among all cell types, macrophages showed the highest expression of both S100A8 (7630 transcripts per million) and S100A9 (8840 transcripts per million). M0, M1, and M2 macrophages were the most common macrophages. We prepared various macrophages and detected M1 biomarkers (CD68, CD86, and CD80) and M2 markers (IL10 and CD206) to verify the successful polarization. Next, we detected S100A8 and S100A9 expression in different types of macrophages. qPCR and Western blotting both showed that S100A8 and S100A9 expression in M1-polarized macrophages was much higher than in M0 and M2 macrophages, indicating that M1-polarized macrophages were the predominant origination of S100A8 and S100A9 in renal tissue (Figure 1). According to these findings, M1-polarized macrophages were focused on in subsequent experiments. 

***COM-treated HK-2 cells promoted S100A8 and S100A9 expression in M1-polarized macrophages***

**Figure 2:**
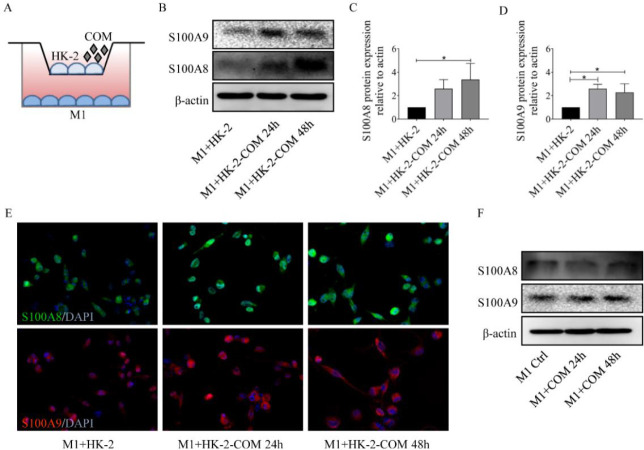
COM-treated HK-2 cells promoted S100A8 and S100A9 expression in macrophages of M1 type. (A) Schematic diagram of cell coculture experiments. (B-D) Western blotting showed that S100A8 and S100A9 expression in M1-polarized macrophages was significantly up-regulated after coculture with COM-treated HK-2 cells. (E) Immunofluorescence analysis showed that COM-treated HK-2 cells promoted S100A8 and S100A9 expression in macrophages of M1 type. (F) Western blotting showed that direct coculture with COM did not affect S100A8 and S100A9 expression in M1-polarized macrophages (*P<0.05)

We conducted cell coculture experiments to simulate the microenvironment of CaOx stones. Western blotting revealed that after coculture with COM-treated HK-2 cells, M1-polarized macrophages showed higher expression of S100A9 and S100A8. This result was further confirmed via an immunofluorescence assay. However, direct coculture with COM had no effect on the expression of these two calgranulins in M1-polarized macrophages (Figure 2). These findings indicated that something from COM-treated HK-2 cells played an important role in up-regulating S100A8 and S100A9 expression in macrophages of M1 type.

***COM could promote HK-2 cells to secrete IL6***

**Figure 3:**
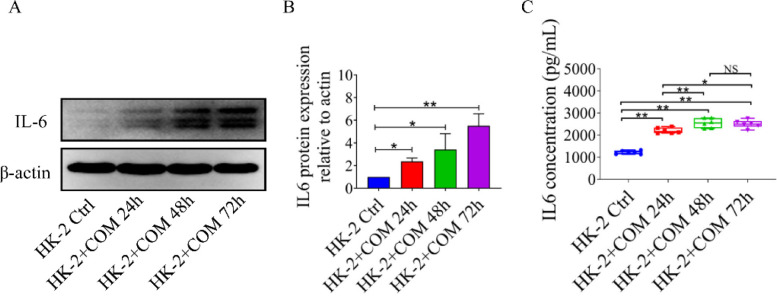
COM could significantly promote HK-2 cells to secrete IL6. (A, B) Western blotting showed that COM could promote the expression of IL6 in HK-2 cells. (C) ELISA revealed that COM could promote the secretion of IL6 in HK-2 cells. (*P<0.05, **P<0.01, NS: Not significant)

The mechanism by which COM-treated HK-2 cells promoted S100A8 and S100A9 expression in M1-polarized macrophages was unknown. We conducted a literature search to explore the mRNA profiles in COM-treated HK-2 cells. Reassuringly, Wang *et al*. have once reported corresponding profiles, and we found that IL6 was one of the four up-regulated mRNAs in COM-treated HK-2 cells (14). As we know, IL6 is an important cytokine that regulates the biological functions of many immune cells (including macrophages). Therefore, IL6 was chosen for further study. We further confirmed that COM could promote the protein expression and secretion of IL6 in HK-2 cells. In addition, the concentration of IL6 in the supernatant of COM-treated HK-2 cells was approximately 2 ng/ml to 3 ng/ml (Figure 3).

***IL6 could up-regulate S100A8 and S100A9 expression in macrophages of M1 type***

**Figure 4:**
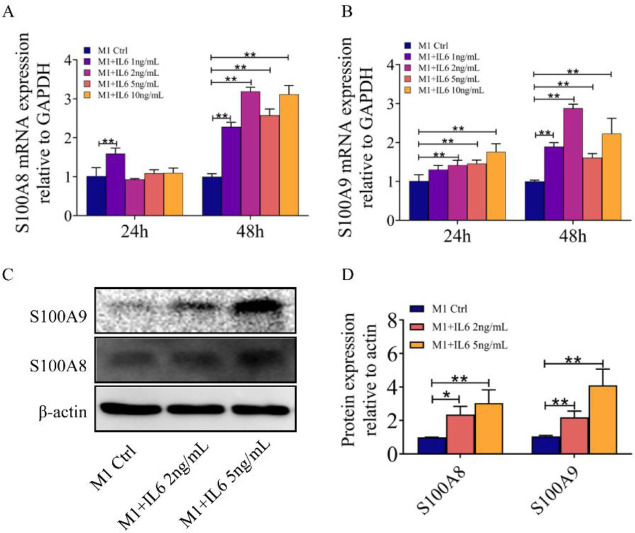
IL6 could up-regulate S100A8 and S100A9 expression in macrophages of M1 type. (A, B) qPCR revealed that M1-polarized macrophages expressed more S100A8 and S100A9 after being treated with IL6. (C, D) Western blotting showed that M1-polarized macrophages expressed more S100A8 and S100A9 after being treated with IL6 (*P<0.05 and **P<0.01)

According to the above findings, we used different doses of IL6 (0 ng/ml, 1 ng/ml, 2 ng/ml, 5 ng/ml, and 10 ng/ml) to culture M1-polarized macrophages. qPCR showed that S100A8 and S100A9 expression was up-regulated in M1-polarized macrophages. This biological effect was most pronounced at 48 hr. Western blotting also revealed similar results, which demonstrated that IL6 could up-regulate S100A8 and S100A9 expression in macrophages of M1 type (Figure 4).

***SC144 kept COM-treated HK-2 cells from up-regulating S100A8 and S100A9 expression in M1-polarized macrophages***

**Figure 5:**
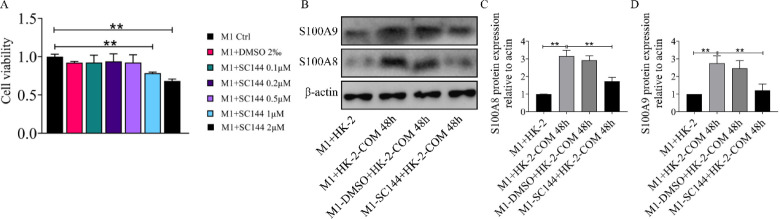
SC144 kept COM-treated HK-2 cells from up-regulating S100A8 and S100A9 expression in M1-polarized macrophages. (A) CCK-8 showed that when the concentration of SC144 reached 1 μM, the viability of M1-polarized macrophages was decreased. (B-D) Western blotting showed that 0.5 μM SC144 kept COM-treated HK-2 cells from up-regulating S100A8 and S100A9 expression in M1-polarized macrophages (*P<0.05 and **P<0.01)

SC144 is a kind of IL6 inhibitor. The CCK-8 assay revealed that when treated with 1 μM SC144 for 48 hr, M1-polarized macrophages showed decreased cellular viability. Accordingly, 0.5 μM SC144 was used to treat M1-macrophages in subsequent coculture experiments. Western blotting showed that 0.5 μM SC144 significantly kept COM-treated HK-2 cells from promoting M1-polarized macrophages to express S100A8 and S100A9, indicating that IL6 from COM-treated HK-2 cells plays an important role in up-regulating S100A8 and S100A9 expression in macrophages of M1 type (Figure 5).

**Table 1:** Primers used in the current study are designed according to human gene sequences

**Gene**	**Species**	**Forward primer**	**Reverse primer**
CD68	Human	CTACTGGCAGAGAGCACTGG	AGTTGAGGGTCCCTGGCT
GAPDH	Human	ACTAGGCGCTCACTGTTCTC	GCCCAATACGACCAAATCCG
CD80	Human	TTTGACCCTAAGCATCTGAAGC	ACCAGCCAGCACCAAGAG
IL-10	Human	TGAAGAATGCCTTTAATAAGCTCC	AGTCGCCACCCTGATGTCT
CD86	Human	CTGCTCATCTATACACGGTTACC	GGAAACGTCGTACAGTTCTGTG
CD206	Human	TTCGGACACCCATCGGAATTT	CACAAGCGCTGCGTGGAT
S100A8	Human	AATTTCCATGCCGTCTACAG	CGCCCATCTTTATCACCAG
S100A9	Human	CGGCTTTGACAGAGTGCAAG	GCCCCAGCTTCACAGAGTAT

## Discussion

With the development of mass spectrometry, proteomic studies have frequently identified calgranulins S100A8 and S100A9 in the kidney stone matrix (6). These two calgranulins belong to the S100 family, which is characterized by approximately 10kDa molecular weight, high solubility in saturated ammonium sulfate, a helix-loop-helix motif, and a high affinity for calcium ions (15). There are 93 and 113 amino acid residues in human S100A8 and S100A9, respectively (16). They can form a stable heterodimer or homodimer *in vivo* and play an important role in inflammation by stimulating the recruitment of leukocytes and inducing the secretion of cytokines (17, 18). In addition, researchers reported that vesicles containing S100A9 from macrophages are powerful in binding crystals and promoting vascular calcification (19). However, how S100A8 and S100A9 participate in kidney stone formation is unknown. 

S100A8 and S100A9 primarily originate from immunocytes, including macrophages and neutrophils (15). According to the single-cell sequencing map from the Single Cell Type Atlas, we found that macrophages were the predominant source of renal S100A8 and S100A9. Correspondingly, macrophages have been found to significantly infiltrate the renal tissue of patients with nephrolithiasis (20, 21). Moreover, we previously observed that positive S100A8 and S100A9 staining was restricted to intravascular cells of normal renal tissue but was distributed in the interstitium of stone renal tissue, which then could be explained by current findings. Among various macrophages, M1-polarized macrophages were the primary subtype expressing S100A8 and S100A9. In addition, COM-treated HK-2 cells could promote the expression of these two calgranulins in M1-polarized macrophages. Therefore, we could make a reasonable conclusion that renal-up-regulated S100A8 and S100A9 in kidney stone patients are predominantly derived from macrophages of M1 type. M1-polarized macrophages are thought to play a pro-inflammatory role in the immune response by secreting inflammatory factors and maintaining inflammation (22). A study reported that induction of M1 polarization could promote renal CaOx crystal deposition in hyperoxaluria rats (23). It is unclear whether S100A8 and S100A9 from M1-polarized macrophages play proinflammatory and lithogenic roles in nephrolithiasis. We are constructing S100A8 and S100A9 knockout mice to explore this question. Preliminary results revealed that knocking out S100A8 and S100A9 could decrease renal CaOx crystal deposition in hyperoxaluria mice (data have not been published).

An increasing number of studies indicate that stone formation involves not only supersaturated precipitation of urine but also the interaction between cells and urinary crystals (24-26). The role of crystal-cell inflammation in stone formation has attracted increasing attention in recent years (27). According to mRNA expression profiles presented by Wang *et al*. (14), we identified IL6 as an important up-regulated cytokine in HK-2 cells treated with COM. Previous studies have also found that renal expression of IL6 was elevated in hyperoxaluria mice (28). The role of IL6 in the inflammatory response during kidney stone formation is not clear. In our study, we demonstrated that IL6 could promote S100A8 and S100A9 expression in M1-polarized macrophages. Similarly, researchers reported that IL6 up-regulated mRNA levels of S100A8 and S100A9 in THP-1 cells by activating the advanced glycation end-product signal transduction pathway (29). Therefore, we hypothesized that COM-treated HK-2 cells might promote S100A8 and S100A9 expression in M1-polarized macrophages by secreting IL6. Further SC144 blocking experiments confirmed this supposition. These findings revealed a cascade immune response involving IL6, S100A8, and S100A9 between CaOx crystals, renal tubular epithelial cells, and macrophages.

There were several limitations in our study. First, all experiments were conducted *in vitro. In vivo *studies are needed in the future to confirm current findings. Second, although IL6 was found to significantly promote S100A8 and S100A9 expression in macrophages, there might also be other important regulators. Finally, the mechanism underlying how IL6 regulates the expression of S100A9 and S100A9 requires further exploration.

## Conclusion

M1-polarized macrophages were the predominant cell type expressing S100A8 and S100A9 in the kidneys of CaOx stone patients. CaOx crystals can promote renal tubular epithelial cells to secrete IL6 to up-regulate S100A8 and S100A9 expression in macrophages of M1 type.

## Authors’ Contributions

SF and JKH designed the experiments; WQ performed experiments and collected data; WQ, CXL, and ZJQ discussed the results and strategy; JKH supervised, directed, and managed the study; WQ, ZJQ, CXL, SF, and JKH finally approved the version to be published.

## Ethical Statement

This article does not contain any studies with human participants or animals performed by any of the authors.

## Conflicts of Interest

The authors declare that they have no conflicts of interest.
